# Health Tract, No. 261

**Published:** 1866-09

**Authors:** 


### BOILS

Are Nature's method of avoiding or curing aisease, A Boil begins with a hard lump, which increases in size, heat and painfulness for about seven days; then it begins to "point," and a yellow speck at the top is seen. This spreads and finally 'breaks,' discharging more or less blood and matter for two or three days when the "core" comes out, the pain ceases, the hollow left is by degrees filled up with new flesh and in about fourteen days from the beginning, the patient is well, at least of that one! But sometimes a second one breaks out before the first one is well; or a dozen or more appear in various parts of the body in various stages.

Job was covered with boils. The Romans designated them by the Latin word which means to "make mad," or ill-natured. Only saints can be serene when a boil is coming to a point.— The old and young, the vigorous and the weakly, all are exposed to them; but with this difference: in the robust they run their course in about fourteen days and get well of themselves. In persons of feeble constitution a boil becomes a carbuncle, which is many boils springing up near to-gether. These often prove fatal, especially with those who use ardent spirits. The general treatment is to call in a surgeon and have it cut to the bone in a cross. In every case keep the parts moist all the time by a poultice of sweet milk and stale bread; nothing better, safer or more handy can be used; it remains moist longer than most others, and is easily softened and removed preparatory to renewals, which should be made thrice a day.

Boils are the result of impure blood, made so by imperfect digestion: or an excess of bile, owing to a torpid liver or the want of sufficient out-of-door exercise. They are not a sign of health, but that nature is carrying on a healthful process. A felon or whitlow, is a boil formed on the bone under the whit-leather or broad tendons, which are so impervious that the yellow matter can not be worked out through them; hence, if not promptly cut down upon, to let out the yellow matter, it must get well by the slow and fearfully painful process of re-absorbtion. As to a common boil, all that should be done is to render the process of cure less painful by moist poultices, by living on coarse bread, ripe raw fruits, berries and tomatoes in their natural state, using no sweets, oils, meats or spirits. If the constitution is feeble, beef-soups and other nourishing food is necessary. Be out of doors; keep the skin clean and have the bowels act freely every day. The Saxon name "Bile" is the best term, because it is really nature's process of discharging extra bile from the system, with other hurtful humors which ought to be out of it. If boils follow fever or other disease, it shows that they were not treated with sufficient activity.

